# The mediating effect of going concern and corporate reporting in the relationship between corporate governance and investor confidence in financial institutions^☆^^☆☆^

**DOI:** 10.1016/j.heliyon.2023.e20447

**Published:** 2023-09-29

**Authors:** Paul Hammond, Mustapha Osman Opoku

**Affiliations:** a Department of Entrepreneurship and Business Sciences, University of Energy and Natural Resources, Sunyani, Ghana; b Catholic University of Ghana, Sunyani Faculty of Economics and Business Administration, Ghana

**Keywords:** Corporate governance, Corporate reporting, Financial institutions, Going concern, Investor confidence

## Abstract

This study investigates the mediating role of going concern and corporate reporting on the relationship between corporate governance and investor confidence in financial institutions. The study employed Partial Least Squares Structural Equation Modeling (PLS-SEM) in SmartPLS 3 to analyze the data. The data for the study was collected from financial statements of selected commercial banks in Ghana, Nigeria and South Africa. The results indicate that corporate reporting partially mediates the interrelationships between corporate governance, going concern, and investor confidence. Conversely, there is neither mediation effect of going concern on the association between corporate reporting and investor confidence, nor between corporate governance and investor confidence. The results of the study have practical implications for financial institutions looking to maintain investor confidence and promote financial stability. The results also have policy implications for policymakers and regulators that oversee financial institutions. Knowledge in the field of corporate reporting and governance theoretically also is extended by highlighting the importance of transparency and disclosure in corporate reporting practices. In all, this study contributes to the literature on corporate governance and reporting by providing new insights into the mechanisms by which corporate reporting and going concern impact corporate governance and investor confidence in financial institutions.

## 1 Introduction

Investor confidence in financial institutions has taken a hit since the 2008 financial crisis and subsequent corporate scandals [1,2]. These incidents have underlined the importance of ensuring the credibility and accuracy of corporate reporting, particularly with regard to the going concern status of financial institutions in business [3,4].

The going concern status of financial institutions is of utmost significance because these institutions play an essential role in the economy of the entire world and are frequently regarded as too crucial to fail [5,6]. According to Altman, Hotchkiss [7], recent high-profile company collapses, such as Enron, WorldCom, and Lehman Brothers, which had enormous effects on the overall structure of the global financial system, have brought to light the significance of the concept of a going concern. A corporation is considered to be in a going concern position if it is able to maintain its current level of operations into the foreseeable future without the need for significant reorganisation or liquidation. The concept of a going concern is essential for investors and other stakeholders in the financial industry, as it demonstrates that a business is financially stable and able to fulfil its commitments.

Another essential aspect in the financial business is investor confidence, which refers to investors' belief in a company's capacity to deliver on its promises and satisfy its financial responsibilities [8]. Several factors affect investor confidence, including a company's financial performance, regulatory compliance, transparency, accountability, communication, and economic situations [9]. Investor confidence has a direct impact on a company's capacity to raise cash and obtain credit and thus on its ability to develop and prosper. Investor confidence in financial institutions has been considerably weakened as a result of the global financial crisis and other corporate scandals [10]. This lack of confidence has resulted in decreased investment and liquidity, which has the potential to destabilise the financial system. Given these trends, it is critical to explore the factors influencing investor trust in financial institutions.

Therefore, policymakers, regulators, and financial institutions must understand and increase their knowledge of the elements that influence investor trust in financial institutions. The purpose of this research is to investigate the role that mediators, such as corporate reporting and going concern, play in the connection between investor confidence and corporate governance. The results of the study will contribute to a deeper understanding of the elements that determine the level of confidence that investors have in financial institutions. The study will provide a better understanding of the factors that influence investor confidence in financial institutions. In this study, we also examined the relationships between corporate reporting, corporate governance, going concern, and investor confidence in financial institutions.

This study is significant for many reasons. First, research has acknowledged the crucial role of the financial sector in driving higher economic growth and promoting development in Africa [11]. Acting as intermediaries, the financial institutions provide avenue for savings and investment, thereby facilitating growth rates of any economy. However, Africa's financial sector has been relatively weak, contributing to a lower savings rate compared to other developing countries [12]. Therefore, investigating and implementing a mechanism to strengthen the financial sector on the continent becomes imperative.

Moreover, African leaders have taken decisive action to boost trading activities within the continent, leading to the establishment of the African Continental Free Trade Agreement (AfCFTA). The role of financial institutions in this endeavour is paramount. They are expected to play a critical role in financing infrastructural projects that facilitate the movement of goods and services, as well as supporting efficient transactions [13,14]. Additionally, local firms will require financial backing to expand production, improve product quality, invest in advertising, and facilitate exports. As a result, the support provided by financial institutions will be instrumental in driving economic growth and fostering increased trade and prosperity within Africa. Thus, any effort to promote functioning financial sector is highly recommended.

Finally, recent developments in the financial sector have brought to light instances of commercial banks and other financial institutions facing insolvency or having their licences revoked due to non-compliance with their obligations [15,16]. These incidents have been attributed to weak corporate governance practises, leading to a decline in investor confidence, particularly in countries with leading-performing economies such as Ghana, South Africa, and Nigeria. The dwindling confidence of investors underscores the urgency for African countries to strategize and implement measures that restore trust in the financial sector. One way to achieve this is by examining the role of going-concern assessments and implementing sound financial reporting practises. These measures will instill transparency, accountability, and reliability, ultimately bolstering investor confidence.

Despite the critical nature of these issues, there remains a significant gap in research and studies focused on financial institutions on the African continent. Therefore, conducting such a study as this into the state of financial institutions in Africa is crucial. Addressing the challenges identified and exploring the potential of going-concern assessments and sound financial reporting will not only restore investor confidence but also significantly contribute to the overall economic growth and development of the continent. By prioritizing the enhancement of corporate governance practises and fostering trust between investors and financial institutions, Africa can pave the way for a resilient and thriving financial sector that will drive sustainable economic progress in the long term.

The remaining parts of the research are organised as follows: Section 2 provides a theoretical and empirical review of relevant literature; Section 3 provides the methodologies and data for the study; Section 4 presents the results of the research; Section 5 discusses the findings; and Section 6 concludes the study by providing practical, policy, and theoretical implications as well as the direction for future research.

## 2 Literature review

### 2.1 Theoretical review

The study is grounded on signalling theory and stakeholder theory.

#### 2.1.1 Signaling theory

Signalling theory suggests that financial institutions use their financial reporting to signal their financial health and performance to investors [[17], [18], [19]]. According to this theory, financial institutions with higher quality financial reporting are perceived by investors to be more financially sound and thus are more likely to attract investment.

According to Auronen [20], signalling theory is a theory that comes from the field of economics that explains how parties who have asymmetric knowledge, such as investors and corporations, interact with one another. According to signalling theory, companies use their observable qualities, such as their financial reports and corporate disclosures, to communicate to investors the genuine underlying quality of their businesses [21]. This is due to the fact that investors do not have access to all information regarding the company's actual quality or performance; as a result, they rely on signals to determine the value of the company and to make decisions regarding investments. If a bank publishes reliable financial reports, for instance, that sends a message to potential investors that the bank is solvent and managed well. Similarly, if auditors express doubt about a financial institution's ability to continue as a going concern, that could send a message to investors that the company is struggling financially and could soon go bankrupt [22].

The signalling theory provides an explanation for the role that going concern and corporate reporting play as mediators in the link that exists between investor confidence and the functioning of financial institutions. Financial institutions that have corporate reporting that is of a higher quality have a greater chance of receiving positive going concern opinion from auditors. This view indicates to investors that the financial institution is financially stable and reliable. Because of this, there is a possibility that investor confidence and trust in the financial institution may increase.

#### 2.1.2 Stakeholder theory

The accountability of management to a wide variety of stakeholders with varying interests was the core idea behind Freeman [23] formulation of the stakeholder theory. The interaction between an organisation and the various stakeholders in that organisation is investigated by this theory [24,25]. It is of the view that managers in organisations have a variety of groupings that they are responsible for serving. Investors, depositors, government regulators, and staff are all considered to be “stakeholders” in the context of financial institutions. Financial institutions have a responsibility to these stakeholders to provide accurate and reliable, and they also have a responsibility to act in the best interest of these stakeholders. For instance, financial organisations may place a higher priority on the interests of depositors by making sure that their funds are kept secure, or they may place a higher priority on the interests of employees by ensuring that the workplace is both safe and healthy. In the same vein, investors rely on information provided by financial institutions to make investment decisions. It is the responsibility of financial institutions to ensure the confidence and trust of their investors by providing them with information about the company's performance that is accurate and dependable.

Stakeholder theory is founded on the premise that values are essential and explicit components of business transactions. Therefore, according to Jones, Melis [3], managers are expected to demonstrate a clear understanding of the value they create and the relationships between the organization's important players. Moreover, Freeman, Wicks [26] urged executives to be explicit about their mode of conduct, particularly the types of relationships they require, and to form bonds with stakeholders in order to achieve the desired goals.

In the traditional view of a company, called the shareholder view, the shareholders are the company's owners, and the business has a legal duty to put their interests first and grow their value (Bowie, 2012). On the other hand, stakeholder theory [3] states that there are other parties involved, such as workers, investors, suppliers, government agencies, customers, trade unions, trade associations, political groups, financiers, and communities [25,26]. Even competitors are sometimes counted as stakeholders because they can affect how the company and other stakeholders work. Stakeholder theory says that companies have to take care of not only their shareholders but also their workers, customers, and the community. The theory advocates that the interests of stakeholders should be taken into account when making decisions that affect how well a business does and how long it can stay in business.

According to Mitchell and Cohen [27], the purpose of stakeholder theory is to broaden management's conception of its role and responsibilities beyond profit maximisation activities to include the interests and prerogatives of groups that are not shareholders. Management is held accountable by the firm's stakeholders to carry out the actions and disclose the information deemed essential by those stakeholders. Therefore, managers should take into account the needs of all stakeholders, and managers should integrate the interests of different stakeholders without giving any set of stakeholders clear precedence in order to maximise the welfare of all stakeholders [23,26]. In light of this, financial institutions should make meeting the requirements and addressing the concerns of their stakeholders a top priority, particularly the provision of trustworthy financial data. Therefore, investors are more likely to have faith in financial organisations that put their stakeholders first and whose financial reports are of a higher quality and who receive good going concern assessment from their auditors.

Overall, the signaling and stakeholder theories are relevant to the study of the mediating role of going concern and corporate reporting on investor confidence in financial institutions. According to the stakeholder theory, financial institutions have a responsibility to act in the best interests of their stakeholders, which includes providing accurate and reliable financial information. Signalling theory, on the other hand, proposes that financial institutions use their observable characteristics to signal their quality to investors.

### 2.2 Empirical review and hypotheses

In recent years, substantial studies have been conducted on investor confidence in financial institutions. Many studies have been conducted to investigate the elements influencing investor confidence, such as corporate governance, financial reporting quality, and regulatory compliance [6]. Several studies have been conducted to investigate the link between going concern, corporate reporting, and investor confidence.

Brunelli, Carlino [28] conducted research in the Italian market to determine how investors react to audit reports that contain a going concern modification. This research made use of a technique known as event study in order to investigate the brief event windows experienced by Italian-listed firms between the years 2009 and 2015. The study shed light on how investors respond to audit reports that contain a going concern modification in the market by providing insights gleaned from those reactions. The fact that going concern disclosures were found to have a positive relationship with investor confidence lends credence to the notion that investors rely on this information when choosing how to allocate their capital. In a similar vein, Mohamed, Allini [29] investigated the extent to which the disclosures made by Egyptian companies in their management reports were helpful from the perspective of financial analysts and institutional investors. According to the findings of the study, investors have various perceptions of required and voluntary disclosures, and some voluntary disclosures are viewed as more valuable than mandated disclosures. Thus, Hypothesis 1 proposes that going concern mediates the relationship between corporate reporting and investor confidence.

Corporate governance influences reporting quality and increases investor confidence in effective management [30,31]. A study by Xiaolu, Jieji [31] concluded that excellent corporate governance contributes to the authenticity of the accountability mechanism, the quality of financial information, and the dependability and integrity of the capital market, thereby bolstering investor confidence. According to Mahrani and Soewarno [30], excellent corporate governance ensures the efficient operation of the accountability system and enhances the dependability and quality of corporate data. Muda, Maulana [32] reported that the transition to efficient corporate governance had a positive impact on reporting quality and investor confidence, as well as influencing the future development of companies, and that companies with good corporate governance tend to publish their financial statements quickly as a result of effective internal control. Hence, <b>Hypothesis 2 suggests that going concern also mediates the relationship between corporate governance and investor confidence</b>.

According to Yadav [33], investors are more likely to trust a company's report if it includes the auditor's view on the financial statement's accuracy and fairness. But there are examples of companies that failed even after an audit showed a healthy financial position. It is the obligation of management or the governing bodies of entities to maintain accurate records, compile final accounts, and hire external auditors to review the books. This lends credence to the agency theory, which advocates a transparent accounting information system to increase investor trust. Yadav [33] argues that investors are more likely to trust a company's report if an independent auditor has vouched for its accuracy and fairness. Thus, Hypothesis 3 proposes that corporate reporting mediates the relationship between corporate governance and going concern in financial institutions.

The vast majority of models that are used to forecast whether or not a business will continue to operate are based on information taken from financial statements. Therefore, it is reasonable to deduce that the acceptance of the result of any model depends on the quality of the information included in the financial reports. This is because the quality of the information is directly related to the acceptability of the result. According to the findings of Obiyo and Ezenwa [34], the credibility of the financial report can be determined by examining whether or not the information it provides paints an accurate image of the current situation of the organisation. According to Obiyo and Ezenwa [34], the information reliability of financial statements is improved when company transactions are represented in a manner that is both truthful and fair. The effect of financial ratios on the going concern (or financial distress) of companies and the dependability of such information for users has been demonstrated by previous studies. Hosaka [35] conducted research to investigate the connection that exists between financial ratios and the monetary health of businesses. According to the findings, certain financial parameters, such as liquidity, have a favourable effect on the level of financial difficulty experienced by the company. Therefore, Hypothesis 4 suggests that corporate reporting also mediates the relationship between corporate governance and investor confidence.

## 3 Methods and material

### 3.1 Sample and data collection

This study used data from commercial banks in Ghana, Nigeria, and South Africa for the analysis. These countries were chosen due to their strong performing stock markets in the region and recent financial sector clean-ups that took place. The study specifically concentrated on banking institutions because of their size and distinct characteristics, although the financial sector also includes insurance companies and microfinance enterprises. After the clean-up of the financial sector, Ghana has 23 licensed commercial banks, according to the Bank of Ghana [36]. The Central Bank of Nigeria reported a total of 24 of them being commercial banks as of 2021. In South Africa, the South African Reserve Bank stated that there are 31 banking entities consisting of 18 local banks and 13 local branches of foreign banks. For the study, 15 listed commercial banks from Ghana, 10 from Nigeria, and 10 from South Africa were purposefully selected based on complete data availability for the period under consideration. The study utilised secondary data obtained from the financial statements of these selected banks. The data used in the study were extracted from the websites of the banks and stock exchanges. The financial statements covered a ten-year period from 2011 to 2020, resulting in a total of 350 firm-year observations, with 35 observations per year over the ten-year span.

### 3.2 Data analysis method

The study utilised SmartPls 3 software to analyze the data using Partial Least Squares Structural Equation Modelling (PLS-SEM). The partial least squares structural equation modelling technique is widely used in business and social science research. PLS-SEM permits researchers to investigate and test hypotheses regarding complex relationships between multiple variables [37,38]. PLS-SEM has grown in popularity due to its capacity to manage small sample sizes and non-normal data distributions [37], which makes it particularly useful for financial research.

The model used for the research consisted of four different constructs, which were investor confidence, corporate governance, corporate reporting, and going concern. As a paradigm for mediation testing, we used the going concern and corporate reporting as mediators. These are the constructs and the latent variables used in the study:

Independent Variable (Construct) - Corporate Governance Variables.

Corporate governance is the system of rules, practices, and processes by which a company is directed and controlled. It is designed to ensure that the company is managed in the best interests of its shareholders and other stakeholders. It is represented by:

• CG 1 Number of non-executive board members
• CG 2 Number of female members on the board
• CG 3 The number of board members

Mediating Variables (Constructs):

#### 3.2.1 Going concern

Going concern is a term used to describe the ability of an organization to continue operating in the foreseeable future. When a company is considered to be a going concern, it is assumed that it will be able to meet its financial obligations and continue to operate for the foreseeable future. The variable used for going concern is the z-scores by Altman, Hartzell [39] computed as follows:

zscore = 3.25 + 6.56WCTA + 3.26RETA + 6.72EBTA + 1.05VETL.

where WCTA is defined as the net working capital divided by total assets; RETA is the retained earnings divided by total assets; EBTA is earnings before interest and taxes divided by total assets; and.

VETL is the market value of equity over the book value of debts.

#### 3.2.2 Corporate reporting ratios

Corporate reporting is the process of disclosing information about a company's financial condition and performance to its stakeholders. This information is typically disclosed in the company's annual report, but it can also be disclosed through other channels, such as press releases and social media. The corporate reporting was represented by the following ratios:

• CR 1 Sales Income divided by Total Assets
• CR 2 Interest Received divided by Total Asset
• CR 3 Current Assets divided by current liabilities
• CR 4 Cash and cash equivalents divided by customers' deposits
• CR 5 Customer Deposits divided by Loans and Advances to customers
• CR 6 Net working capital divided by current liabilities

Dependent Variable - Investor Confidence.

Investor confidence is the belief that investors have in the ability of a financial institution to repay its debts and meet its obligations. It is influenced by a number of factors, including the financial institution's financial condition, its corporate governance practices, and the regulatory environment. The variables used for the investor confidence were as follows:

• IC 1 Natural log of the deposits
• IC 2 Natural log of total equities
• IC 3 Natural log of total share capital

We used Chin, Cheah [40] recommendation of two-step strategy to evaluate SEM models, which consisted of first assessing the measurement models and then analysing the structural models. We performed an analysis of the measurement model to assess whether or not the properties of the latent variables were suitable for the model. Due to the fact that all of the latent variables were represented as reflective of the constructs, this research utilised a reflective model. In light of this, the measurement model was evaluated by performing an analysis of the latent variables' reliability, convergent validity, and discriminant validity. Following the establishment of the reliability and validity thresholds, the structural model under consideration was evaluated. The structural model provides insight into how well the hypothesis fits the data.

## 4 Results

### 6.1 Measurement and structural model assessment

The composited reliability, Cronbach's alpha, and Dijkstra-Henseler's rho were the three reliability measures that were employed in order to evaluate the degree to which the latent variables could be relied upon. The degree to which a given collection of variables of a latent concept are consistent internally in their measurement is what we mean when we talk about reliability. According to Henseler, Hubona [41] and Mohajan [42], an adequate reliability measure is one that is greater than 0.7. The reliability values for all three were within the acceptable range as shown in Table 1. Therefore, it can be concluded that the reliability values were appropriate for the study.

**Table 1:** Reliability statistics of latent variables.

	Cronbach's Alpha	rho_A	Composite Reliability	Average Variance Extracted (AVE)
Corporate Governance	0.894	0.919	0.936	0.83
Corporate Reporting	0.933	0.937	0.949	0.758
Going Concern	1.000	1.000	1.000	1.000
Investor Confidence	0.890	1.101	0.926	0.806

The convergent validity of the constructs was tested. According to Refs. [37,38] convergent validity refers to the extent to which indicators of a given construct meet or have a significant amount of variance in common. It is the extent to which one variable positively correlates with another within the same construct. Items must measure the given latent variable and no other latent variable, according to convergent validity. Table 1 also depicted that the Average Variance Extracted value attained was more than 0.5 for each construct. The implication is that the latent construct captures at least 50% of the measurement variance, demonstrating the constructs' appropriateness [37].

The discriminant validity test was the last test that was done on the measurement model. The degree to which one construct differs from other constructs in terms of the degree to which it correlates with other constructs and the degree to which distinctly measured variables indicate only this one construct is referred to as the discriminant validity of the construct Fornell and Larcker [43] introduced a criterion called Average Variance Extracted (AVE) to assess the convergent validity of latent constructs. They suggested that the AVE of each latent construct should exceed the squared correlation between that construct and any other construct in the model. This criterion helps evaluate whether a construct adequately captures the variance attributed to it and discriminates from other constructs in the model. The results in Table 2 show the criterion was met.

**Table 2:** Discriminant validity test using the Fornell-Larker criterion.

	Corporate Governance	Corporate Reporting	Going Concern	Investor Confidence
Corporate Governance	**0.911**			
Corporate Reporting	0.190	**0.87**		
Going Concern	0.074	0.285	**1**	
Investor Confidence	0.310	0.582	0.339	**0.898**

Note: Square roots of AVEs are shown in bold on the diagonal.

Finally, as depicted in Table 3, the Heterotrait-monotrait ratio criterion was successfully satisfied because all values were lower than the recommended cut-off point of 0.85, which Hair Jr, Hult [38] recommended.

**Table 3:** Discriminant validity test using the Heterotrait-monotrait Ratio.

	Corporate Governance	Corporate Reporting	Going Concern	Investor Confidence
Corporate Governance				
Corporate Reporting	0.208			
Going Concern	0.079	0.293		
Investor Confidence	0.337	0.544	0.327	

After establishing the model's reliability, convergent validity, and discriminant validity, we proceeded to examine the structural path that was present in the model. To evaluate the importance of each potential route, a bootstrapping method was utilised. In the bootstrapping technique, 5000 new samples were drawn with replacement and there was a 95% degree of confidence in the results. The precision of the model was evaluated based on the robustness of each structural path, which was calculated using the R^2^ value of the dependent variable [44]. In order for the model to be considered accurate, the R^2^ value must be larger than or equal to 0.1. In addition, Stone-Geiser Q^2^ demonstrates that the endogenous constructs have a predictive relevance. When Q^2^ is greater than 0, it indicates that the model has some predictive reliance. Once again, the Standardised Root Mean Square Residual (SRMR) composite factor model is utilised in order to evaluate how well the model fits the data. A satisfactory model fit is indicated when the SRMR is less than 0.1 [37]. Every one of the indices was achieved.

**Fig. 1:**
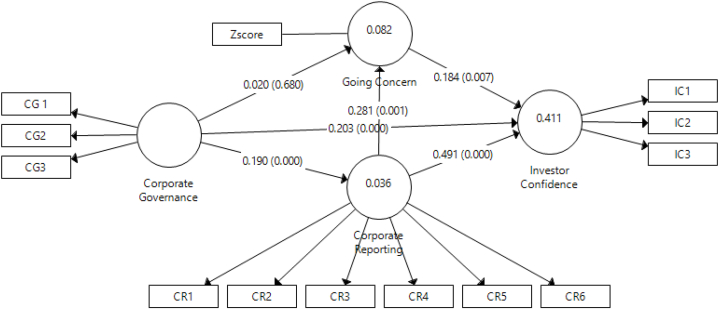
Structural model.

Fig. 1 and Table 4 provide visual and tabular representations of the structural model, illustrating the relationships among the constructs. They provide an overview of how the corporate governance, corporate reporting, going concern and investor confidence constructs are interconnected in the model.

**Table 4:** Direct effect.

	Path	T Statistics	P Values
Corporate Governance → Corporate Reporting	0.190	4.395	0.000
Corporate Governance →Going Concern	0.020	0.413	0.680
Corporate Governance → Investor Confidence	0.203	4.829	0.000
Corporate Reporting → Going Concern	0.281	3.421	0.001
Corporate Reporting → Investor Confidence	0.491	9.774	0.000
Going Concern → Investor Confidence	0.184	2.680	0.007

### 4.2 Mediation effects

Hypotheses 1 and 2 sought to test the mediating effect of going concern on corporate governance and investor confidence; and corporate reporting and investor confidence, respectively. Whilst hypotheses 3 and 4 explored the mediation effects of corporate reporting on the relationships between corporate governance and going concern; and corporate governance and investor confidence.

**Fig. 2:**
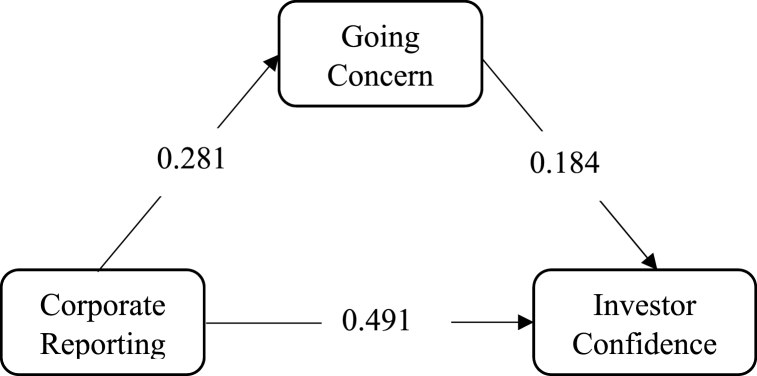
Mediating effect of Going Concern on CR →IC link.

**Fig. 3:**
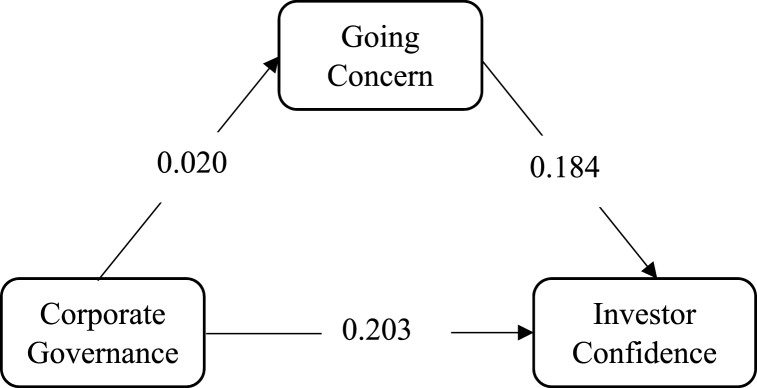
Mediating effect of Going Concern on CG →IC link.

**Fig. 4:**
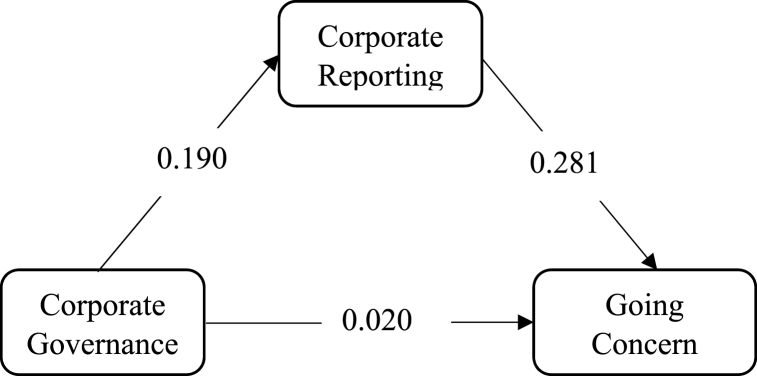
Mediating effect of Corporate reporting on CG→ G C link.

**Fig. 5:**
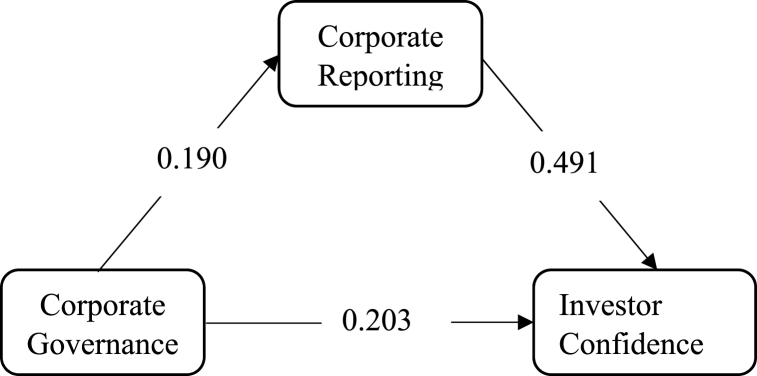
Mediating effect of Corporate reporting on CG→ IC link.

According to Hair, Risher [37], mediation can be tested by finding the Variance Accounted For (VAFs) of the mediators. They recommended that mediating effects should be established if the VAF is greater than 0.2. If the value is higher than 0.2 but less than 0.8, it is considered partial mediation but if the value is more than 0.8, it is recognised as full mediation. The estimated VAFs and links of H_1_, H_1,_ H_1_ and H_4_ are shown in Table 5, Table 6, Table 7, Table 8 and Fig. 2, Fig. 3, Fig. 4, Fig. 5 respectively.

**Table 5:** Mediating effect of Going Concern on CR →IC relationship.

	Path	Estimate
Total Effects	C R → G C	0.281
	G C → I C	0.184
	C R→ IC	0.491
Indirect Effect	C R → IC	0.052
**VAF**	**0.096**	

NB: CG – Corporate Governance, GC - Going Concern, CR – Corporate Reporting (Ratios) and IC – Investor Confidence.

*VAF* = *Indirect Effect/(Direct Effect* + *Indirect Effect)*.

*Indirect Effect = Estimate of (C R → G C) x (G C → I C)*.

**Table 6:** Mediating effect of Going Concern on CG →IC relationship.

	Path	Estimate
Total Effects	C G → G C	0.020
	G C → I C	0.184
	C G → IC	0.203
Indirect Effect	CG → IC	0.004
**VAF**	**0.019**	

Note: VAF = Indirect Effect/(Direct Effect + Indirect Effect).

Indirect Effect = Estimate of (C G → G C) x (G C → I C).

**Table 7:** Mediating effect of Corporate reporting on CG→ G C relationship.

	Path	Estimate
Total Effects	C G → C R	0.190
	C R → G C	0.281
	C G → G C	0.020
Indirect Effect	CG → G C	0.053
**VAF**	**0.726**	

Note: VAF = Indirect Effect/(Direct Effect + Indirect Effect).

Indirect Effect = Estimate of (C G → C T) x (C R → G C).

The result generated from the PLS-SEM for mediation assessment is produced with the VAF calculated in Table 9 below.

**Table 8:** Mediating effect of Corporate reporting on CG→ IC relationship.

	Path	Estimate
Total Effects	C G → C R	0.190
	C R → I C	0.491
	C G → I C	0.203
Indirect Effect	CG → IC	0.093
**VAF**	**0.314**	

Note: VAF = Indirect Effect/(Direct Effect + Indirect Effect).

Indirect Effect = Estimate of (G C → C R) x (C R → I C).

**Table 9:** Summary of mediation results.

Hypothesis	Hypothesised Path	Path Coefficient	T - Statistics	P - Values	VAF	Results
H_1_	CR → GC → IC	0.053	2.089	0.037	0.096	Not Supported
H_2_	CG → GC → IC	0.009	1.782	0.075	0.019	Not Supported
H_3_	CG → CR → GC	0.051	2.437	0.015	0.726	Supported
H_4_	CG → CR → IC	0.079	3.132	0.002	0.314	Supported

NB: CG – Corporate Governance, GC - Going Concern, CR – Corporate Reporting (Ratios) and IC – Investor Confidence.

From Table 5, though there is a statistical significance (p = 0.37) mediation effect of going concern on the relationship between corporate reporting and investor confidence, its variance accounted for (VAF) is below the threshold of 0.2, therefore, there is no mediation effect of going concern on the relationship between corporate reporting and investor confidence. The mediation effect of going concern on the relationship between corporate governance and investor confidence is rejected upright since the VAF is less than 0.2 and the significant value is also more than 0.05.

Corporate reporting, on the other hand, mediates the relationship between corporate governance and the going concern of financial institutions. The result shows that the effect is significant (p = 0.015). Moreover, the VAF is above the 0.2 cut-off point. With a VAF of 0.726, the mediation effect of corporate reporting on the relationship between corporate governance and going concern is considered as partial mediation. The same holds for its mediation role between corporate governance and investor confidence since the effect is significant and VAF is 0.314.

## 5 Discussion

Hypothesis 1 **Going concern mediates the relationship between corporate reporting and investor confidence**.

Although the effect of the mediation role of a going concern on the relationship between corporate reporting and investor confidence was significant, the study was unable to confirm it because variance accounted for (VAF) was below the 0.2 threshold. The outcome indicates that going concern does not mediate the relationship between financial reporting and investor confidence. This result contradicts Brunelli, Carlino [28] and Mohamed, Allini [29], who discovered that going concern has a mediating effect.

The outcome of this analysis leads to the proposition that any efforts aimed at enhancing the company's “going concern” status might yield only minimal or even inconsequential effects on the overarching relationship between corporate reporting practices and investor confidence. This supposition is rooted in the investor behaviour of evaluating a firm's financial performance through a comparative analysis of its financial ratios against those of its peers, thereby forming judgements about its capacity for sustained operation. Consequently, the investor's sense of assurance is fortified when the company demonstrates a robust financial foundation. Therefore, timely dissemination of credible financial reports and assurance of continuity from external auditors increase investor confidence in financial institutions.

Hypothesis 2 Going concern mediates the relationship between corporate governance and investor confidence.

The original statement hypothesizes that going concern plays a mediating role in the relationship between corporate governance and investor confidence. The study found that this hypothesis was not supported at the 95% level of significance (β = 0.009, p = 0.075), thus, did not support the findings of Muda, Maulana [32]. The results showed that going concern did not have a significant impact on investor confidence, even after controlling for the effects of corporate governance. This suggests that investors are not relying on going concern signals when making investment decisions.

Hypothesis 3 Corporate reporting mediates the relationship between corporate governance and Going concern of financial institutions.

The results of the study confirm that corporate reporting mediates the relationship between corporate governance and going concern of financial institutions. The finding supports Yadav [33] that corporate reporting has a major to play in improving going concern status of firms. This result means that by enhancing the quality of corporate reporting, the relationship between corporate governance and going concern could be improved. Thus, the mediation role played by corporate reporting is appreciable. Consequently, the observed mediation role undertaken by corporate reporting emerges as highly commendable. This revelation bears far-reaching implications, elucidating that the meticulous implementation of robust corporate governance measures when coupled with precise and transparent reporting, can synergistically foster a robust going concern position within the realm of financial institutions.

This study's findings serve as a clarion call for a paradigm shift in the approach towards corporate reporting and governance within the financial sector. It underscores the need for institutions to prioritize and invest in refining their reporting practices to align with the highest standards of transparency, accuracy, and comprehensiveness. By doing so, financial entities can enhance their capacity to navigate through challenges and uncertainties, thereby bolstering their resilience in the face of adverse conditions. As the financial landscape continues to evolve, these findings underscore the imperative of holistic and collaborative strategies that encompass both governance enhancements and reporting excellence, ultimately paving the way for a more secure and sustainable financial future.

Hypothesis 4 Corporate reporting mediates the relationship between corporate governance and investor confidence.

The study's findings support the findings of Obiyo and Ezenwa [34] as well as the hypothesis that corporate reporting mediates the link between investor confidence and corporate governance. According to this partial mediation role of corporate reporting, the relationship between corporate reporting and investor trust is said to be strengthened by improving the quality of financial information. This implies that financial institutions that produce high-quality financial reports are more likely to have higher levels of investor trust. The finding also emphasizes the importance of adhering to standard reporting criteria and delivering financial statements on time and in the appropriate format. This is because investors are more likely to trust financial institutions that comply with these standards and that provide them with timely and accurate information. Therefore, in order to attract and retain investors, financial institutions implementing recommended corporate governance should make an effort to adhere to the standard reporting criteria. Thus, managers should make an effort to produce financial statements that meet the appropriate standard and deliver them to shareholders on schedule. Investors' trust and confidence in financial institutions are bolstered when yearly reports with auditors' opinions are delivered on schedule and in the appropriate format.

## 6 Conclusion

The results of this study suggest that corporate reporting plays a partial mediating role in the relationship between corporate governance and going concern, as well as investor confidence. This means that corporate reporting can partially explain the relationship between corporate governance and going concern, as well as the relationship between corporate governance and investor confidence. However, the mediation effect of going concern on the relationship between corporate reporting and investor confidence is weak, and the mediation effect of going concern on the relationship between corporate governance and investor confidence is not supported.

The study findings indicate that corporate reporting can provide investors with information about the financial health of a financial institution, which can help them to assess the institution's ability to continue as a going concern. Again, corporate governance can also influence the quality of corporate reporting, as well as the transparency and reliability of the information that is disclosed. Lastly, investor confidence can be influenced by both corporate reporting and corporate governance. Investors are more likely to be confident in financial institutions that have high-quality corporate reporting and strong corporate governance practices.

The findings of the study have implications for financial institutions, particularly for those institutions that are working to maintain investor confidence and sustain going concern. According to the findings, efficient corporate reporting appears to be critical for ensuring that stakeholders are provided with sufficient information regarding the financial situation of a company. Financial institutions have the ability to boost investor confidence, eliminate uncertainty, and build trust in the financial sector by providing information that is both transparent and accurate on the financial sector. Furthermore, the findings imply that policymakers should encourage financial institutions to adopt transparent and effective corporate reporting practices, as this is vital to maintaining investor confidence and supporting financial stability. In addition, regulators should ensure that financial institutions comply with the laws and standards for reporting in order to promote transparency and fairness of financial information. In addition, the findings support the application of the signalling theory and the stakeholder theory in the setting of financial institutions in order to comprehend the mediating role that corporate reporting plays and the influencers of investor confidence. In all, the findings make a contribution to the existing body of research on the significance of transparency and disclosure in the context of corporate governance and offer fresh perspectives on the part that corporate reporting plays in mediating the connection between corporate governance and financial stability.

Further studies are imperative, as this study focused on the mediation effects of corporate reporting and going concern on investor confidence. Future research could investigate the moderating effect of other factors, such as corporate social responsibility practices and firm size, on the relationship between investor confidence and financial sector growth. By conducting future studies in these areas, researchers could have a deeper understanding of the factors that contribute to investor confidence in financial institutions and provide information for financial institutions and policymakers to improve financial stability and sustainability.

## Data availability statement

Data will be made available on request.

## Ethical approval

Not applicable.

## Ethics approval and consent to participate

Not applicable.

### Funding

The authors did not receive any funding for this study.

## Research involving human participants and/or animals

Not applicable.

## Informed consent

The study did not rely on data or participants that consent was needed.

## Consent to publish

The study did not rely on data/methods/images/figures/participants that consent was needed to publish.

## CRediT authorship contribution statement

**Paul Hammond:** Conceptualization, Formal analysis, Methodology, Writing – original draft, Writing – review & editing. **Mustapha Osman Opoku:** Investigation, Supervision.

## Declaration of competing interest

The authors declare that they have no known competing financial interests or personal relationships that could have appeared to influence the work reported in this paper.
